# Computational Analysis and Experimental Data Exploring the Role of Hesperetin in Ameliorating ADHD and SIRT1/Nrf2/Keap1/OH-1 Signaling

**DOI:** 10.3390/ijms25179284

**Published:** 2024-08-27

**Authors:** Hatem I. Mokhtar, Noha M. Abd El-Fadeal, Rehab M. El-Sayed, Ann Hegazy, Mohamed K. El-Kherbetawy, Ahmed G. Hamad, Mohamed H. ElSayed, Sawsan A. Zaitone

**Affiliations:** 1 Department of Pharmaceutical Chemistry, Faculty of Pharmacy, Sinai University-Kantara Branch, Ismailia 41636, Egypt; 2 Medical Biochemistry and Molecular Biology Department, Faculty of Medicine, Suez Canal University, Ismailia 41522, Egypt; 3 Department of Pharmacology & Toxicology, Faculty of Pharmacy, Sinai University—Arish Branch, Arish, 45511, Egypt; 4 Department of Clinical Pathology, Faculty of Medicine, Suez Canal University, Ismailia 41522, Egypt; 5 Department of Pathology, Faculty of Medicine, Suez Canal University, Ismailia 41522, Egypt; 6 Department of Anatomy and Embryology, Faculty of Medicine, Mansoura University, Mansoura 35516, Egypt; 7 Department of Physiology, Faculty of Medicine, Ain Shams University, Cairo 11757, Egypt; 8 Department of Pharmacology & Toxicology, Faculty of Pharmacy, Suez Canal University, Ismailia 41522, Egypt; szaitone@ut.edu.sa

**Keywords:** ADHD, hesperetin, hippocampal degeneration, mouse, SIRT1/Nrf2/Keap1/OH-1

## Abstract

Attention deficit hyperactivity disorder (ADHD) manifests as poor attention, hyperactivity, as well as impulsive behaviors. Hesperetin (HSP) is a citrus flavanone with strong antioxidant and anti-inflammatory activities. The present study aimed to test hesperetin efficacy in alleviating experimental ADHD in mice and its influence on hippocampal neuron integrity and sirtuin 1 (SIRT1) signaling. An in silico study was performed to test the related proteins. Groups of mice were assigned as control, ADHD model, ADHD/HSP (25 mg/kg), and ADHD/HSP (50 mg/kg). ADHD was induced by feeding with monosodium glutamate (0.4 g/kg, for 8 weeks) and assessed by measuring the motor and attentive behaviors (open filed test, Y-maze test, and marble burying test), histopathological examination of the whole brain tissues, and estimation of inflammatory markers. The in-silico results indicated the putative effects of hesperetin on ADHD by allowing the integration and analysis of large-scale genomic, transcriptomic, and proteomic data. The in vivo results showed that ADHD model mice displayed motor hyperactivity and poor attention in the behavioral tasks and shrank neurons at various hippocampal regions. Further, there was a decline in the mRNA expression and protein levels for SIRT1, the erythroid 2-related factor-2 (Nrf2), kelch like ECH associated protein 1 (Keap1) and hemeoxygenase-1 (OH-1) proteins. Treatment with HSP normalized the motor and attentive behaviors, prevented hippocampal neuron shrinkage, and upregulated SIRT1/Nrf2/Keap1/OH-1 proteins. Taken together, HSP mainly acts by its antioxidant potential. However, therapeutic interventions with hesperetin or a hesperetin-rich diet can be suggested as a complementary treatment in ADHD patients but cannot be suggested as an ADHD treatment per se as it is a heterogeneous and complex disease.

## 1. Introduction

Attention deficit hyperactivity disorder (ADHD) is the third most common mental disorder affecting 3.4% of children worldwide [1,2], with symptoms like struggling to focus, lacking self-controlled behaviors, suffering impulsive behaviors, and demonstrating excessive activities [3]. Moreover, ADHD adults have increased risks for substance use and employment difficulties [4]. ADHD etiology includes gestational, perinatal, and genetic factors. For example, premature birth leads to altered neurogenesis and diminished cortical expansion [5]. Importantly, preterm children show elevated inflammatory molecules that are considered associated with a higher risk of ADHD development [6].

Indeed, neurotransmitter systems have been reported in ADHD [7]. Evidence from brain imaging studies has shown that brain dopamine neurotransmission is disrupted in ADHD [5,6,7,8] and that these deficits may underlie the core symptoms of inattention [8], and impulsivity [8,9,10,11,12]. A body of evidence regarding the dopamine system, reinforcement mechanisms, and ADHD endorses the application of understanding neurobiological mechanisms of reinforcement to the problem of altered reinforcement sensitivity in ADHD [13]. Furthermore, the noradrenergic system is linked to the modulation of higher cortical functions such as alertness, attention, and executive function. Activation of the noradrenergic system strongly affects the performance of attention, which is a cognitive function known to be deficient in ADHD [14].

The growing awareness of the possible persistence of ADHD impairment beyond childhood and adolescence has resulted in increased pharmacotherapy of ADHD in adults. There are some pharmacological interventions for ADHD including stimulant drugs (including norepinephrine and dopamine reuptake inhibitors) such as methylphenidate and dexamphetamine, and non-stimulant drugs (including α2 receptor agonists and selective norepinephrine reuptake inhibitors) such as atomoxetine. However, non-pharmacological interventions include behavior management and cognitive therapy for the child and parental training. The prolonged duration of the use of stimulants and the overall increment in its use raised global concerns about them.

Flavonoids are natural products, and hesperetin is an active ingredient in citrus fruits and a rich area for studying [15]. Multiple pharmacological activities have been reported for hesperetin, such as anti-inflammatory, antioxidant [16], anticancer [17], anti-fibrotic, and immune regulation [18]. Hesperetin activates cellular protective mechanisms, such as nuclear factor erythroid 2-related factor-2 (Nrf2) and hemeoxygenase-1 (HO-1), which are known to play a role in cellular survival in the face of oxidative stress [19]. One study indicated that hesperetin mitigates lipopolysaccharide-induced gliosis in the cortex and hippocampus of mice brains and diminishes the pro-inflammatory activation of nuclear factor-κB (NFκB), expression of interleukin-1β (IL-1β), and tumor necrosis factor-α (TNF-α) [20].

Sirtuins (SIRT) are histone deacetylases that have roles in regulating crucial metabolic pathways. SIRT1 is the most recognized and extensively studied [21]. It is a key mediator in metabolism and, when activated, is considered beneficial in mitigating oxidative stress [22]. Lacking SIRT-1 is harmful to cells and leads to a reduction in viability [23]. SIRT1 was investigated for its therapeutic role in neurologic disorders and was reported to have strong anti-inflammatory activity via the inhibition of NFκB [21]. Cicek et al. asserted that the SIRT1 level was significantly lower in patients with ADHD and is correlated with the severity of cognitive functions [24].

This study aimed to employ bioinformatic tools to suggest the mechanism of the putative protection provided by hesperetin against experimentally induced ADHD in mice and to indicate the signaling pathway that may assist this effect. Hence, we examined the ability of hesperetin to improve the brain’s SIRT1/Nrf2/Keap1/HO-1 signaling.

## 2. Results

### 2.1. Bioinformatic Results Indicating the Relation between the Target Proteins

**Figure 1:**
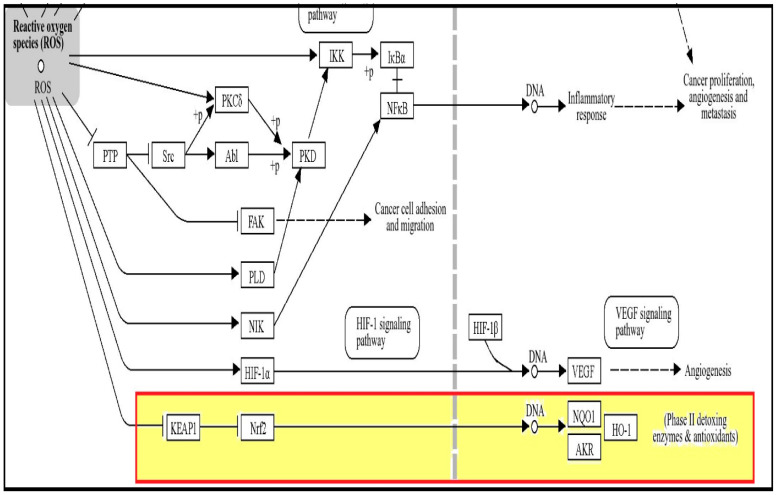
ROS signaling pathway. Elevated ROS production is linked to DNA damage that can result in Keap1/Nrf2/HO-1 pathway of phase II detoxing enzymes and antioxidants.

Using the KEGG pathway database, we searched for the Nrf2/Keap1/heme-oxygenase-1 signaling pathway. We found the proteins’ pathway in the “chemical carcinogenesis—reactive oxygen species” (map05208). We found the proteins in the ROS signaling pathway, indicating the antioxidant action of hesperetin as seen in Figure 1.

**Figure 2:**
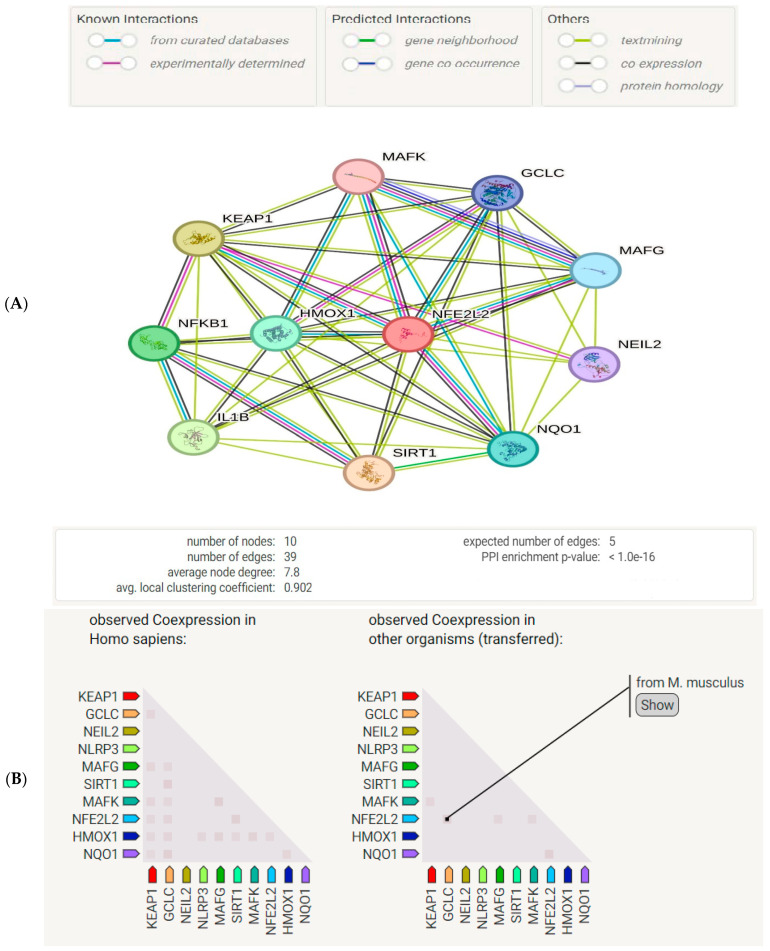
(**A**) A protein–protein interaction network diagram with nodes representing proteins and edges representing the interaction between them. It has significantly higher possible interactions than expected for a group of proteins selected randomly from the genome with the same size and distribution. This enrichment demonstrates that the proteins are correlated with a *p*-value < 1.0 × 10^−16^. NFE2L2, Nrf2; SIRT1, NAD-dependent protein deacetylase sirtuin-1; NEIL2, NFKB nuclear factor NFκB p105 subunit endonuclease 8-like 2; GCLC, glutamate-cysteine ligase catalytic subunit; MAFG, transcription factor MafG; MAFK, transcription factor MafK; NQO1, NAD(P)H dehydrogenase [quinone] 1. (**B**) Gene co-expression of studied proteins generated by STRING db. Co-expression scores are dependent on the pattern of RNA expression and on protein co-regulation as specified by ProteomeHD.

We also used the STRING database to discover protein-protein interactions (PPI) among the studied proteins. A network of nodes and edges was generated representing the proteins and the interactions between them, as shown in Figure 2A. A gene co-expression map was also created using the STRING database for the studied proteins. As shown in Figure 2A, some proteins show co-expression. The hesperetin target genes were retrieved from PubChem and DrugBank db. The genes related to ADHD were searched for in the DisGeNet database (Figure 2B).

**Figure 3:**
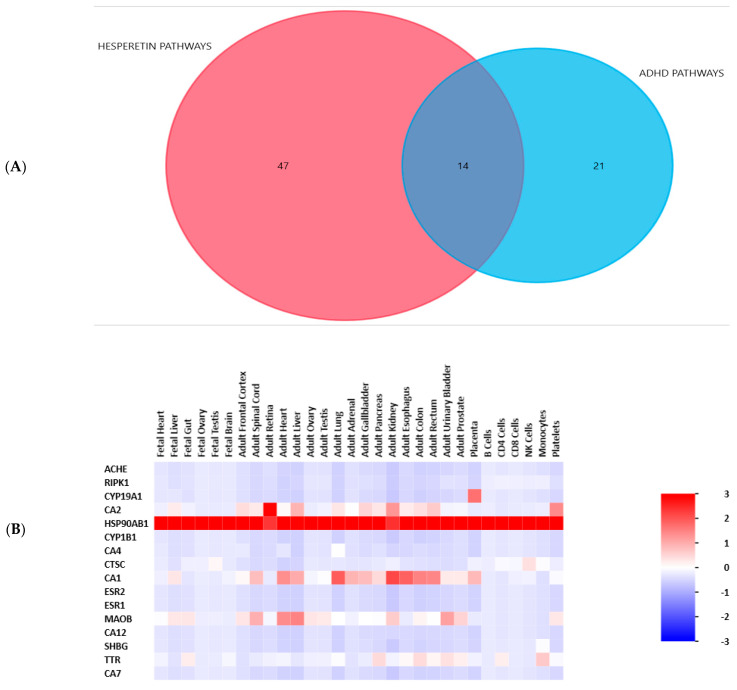
(**A**) A Venn diagram showing the pathways related to hesperetin and to ADHD and the overlapping pathways between them. The diagram was created by the FunRich 3.1.3 bioinformatic tool. (**B**) A heatmap showing the pattern of gene expression of hesperetin target genes. The diagram was created by the FunRich 3.1.3 bioinformatic tool. In the color code, numbers typically range from 3 down to −3, where the values correspond to the degree of enrichment (positive values) or depletion (negative values).

A Venn diagram was created to show the common pathways between hesperetin and ADHD genes. The UniProt ID of each gene was retrieved from the UniProt database, then in the KEGG database, these IDs were converted to KEGG IDs through an ID converter. The related pathways were then searched for in KEGG. Among the 61 KEGG pathways of hesperetin and the 35 KEGG pathways of ADHD genes, 14 pathways were common between them, as shown in Figure 3A. A heat map was created to clarify the patterns of expression of hesperetin genes as shown in Figure 3B.

**Figure 4:**
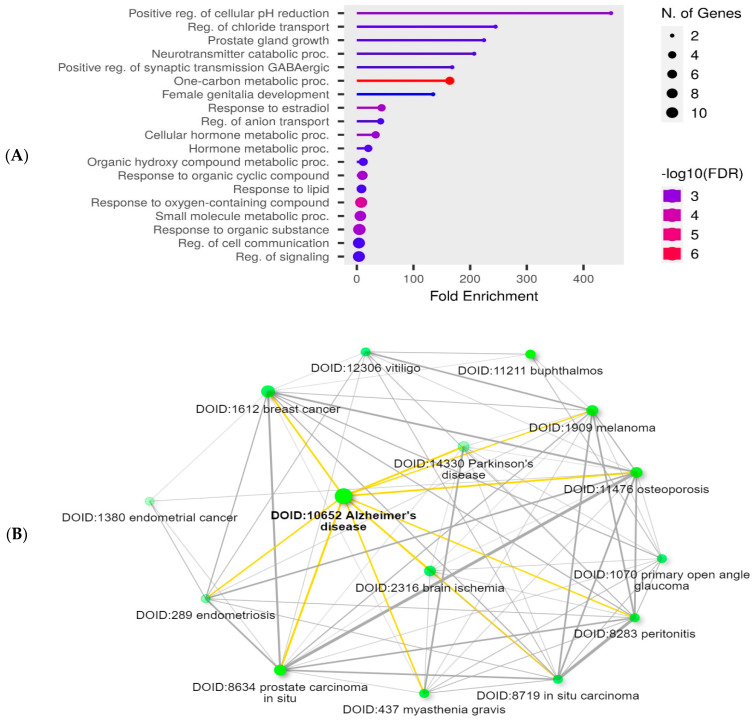
(**A**) Lollipop chart showing the top 20 pathways of GO biological process enrichment analysis of hesperetin target genes. The chart was created with the ShinyGo 0.80 software bioinformatic tool. (**B**) A network displays the relationship between the enriched pathways of the hesperetin target genes according to the Disease Alliance database. Two pathways (represented by nodes) are joined if they share ≥20% of genes. The dark nodes indicate significant enrichment of the gene sets. Larger nodes symbolize larger sets of genes. The thick edges denote more overlapping between these genes. This network was created with the ShinyGo 0.80 software bioinformatic tool.

Functional enrichment analysis was performed for hesperetin target genes using the ShinyGo 0.80 bioinformatic tool. A lollipop chart was created to show the top 20 pathways of GO biological processes related to hesperetin target genes (Figure 4A). In addition, a network was created to show the top 15 diseases related to hesperetin target genes, with brain ischemia, Alzheimer’s disease, and Parkinson’s disease present on the list (Figure 4B).

### 2.2. Effect of Hesperetin on OFT Behaviors

**Figure 5:**
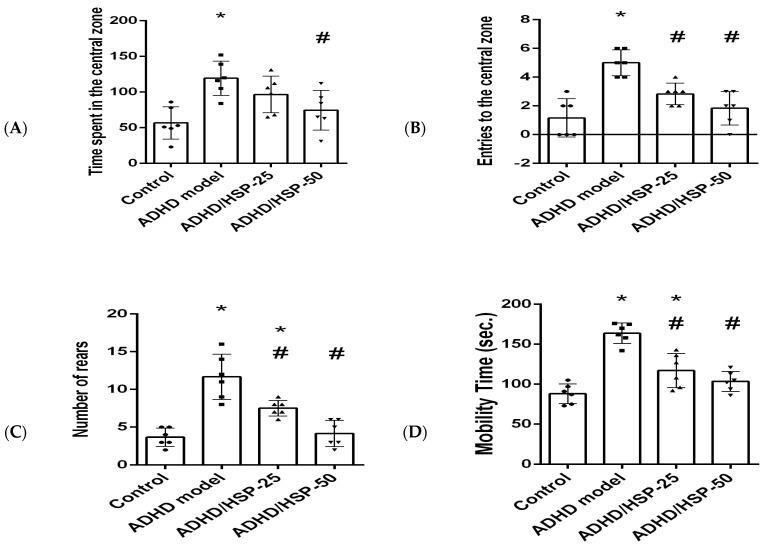

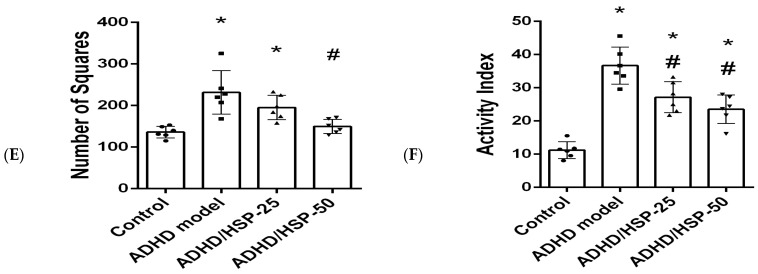
Open field behaviors. (**A**) Times spent in the central zone, (**B**) entries to the central zone, (**C**) rears, (**D**) mobility time (sec), (**E**) number of crossed squares, and (**F**) activity index. Data are mean ± SD. *, #: Versus control, and ADHD model at *p* < 0.05.

In the current study, the ADHD group showed different behavior patterns than the control mice. ADHD mice spent a longer time and performed greater entries to the central zone (Figure 5A,B) and a greater number of rears (Figure 5C) than the control mice. The ADHD/HSP-50 group showed a significantly shorter time spent in the central zone. Both ADHD/HSP-25 or ADHD/HSP-50 groups showed dose-dependent decreases in the number of entries registered in the central zone and the number of rears compared to the ADHD group. In addition, mice in the ADHD group showed longer mobility duration in the testing session (Figure 5D). Both ADHD/HSP-25 and ADHD/HSP-50 groups showed significant decreases in mobility duration.

Figure 5E,F demonstrates the number of crossed squares and the activity index recorded in the OFT, which were greater in the ADHD model group than the control group. The ADHD/HSP-50 group displayed a significant decline in the crossed squares (Figure 5E). Both ADHD/HSP-25 or ADHD/HSP-50 groups showed significant declines in the activity index calculated for the mice in comparison to the ADHD group (Figure 5F).

**Figure 6:**
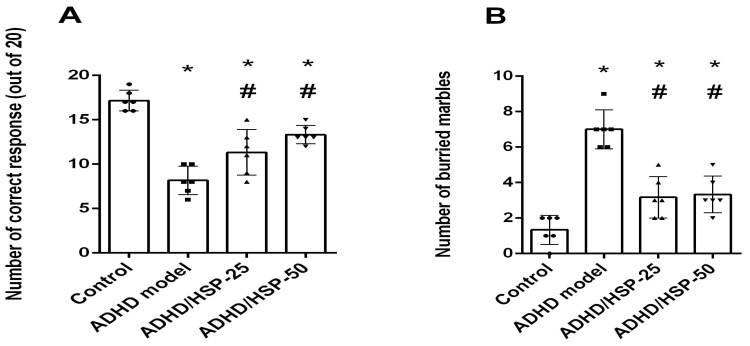
Y-maze and marble-burying behaviors. (**A**) Y-maze test and (**B**) marble burying test. Data are mean ± SD. *, #: Versus control, and ADHD model, at *p* < 0.05.

The results obtained from the Y-maze test demonstrated that mice in the ADHD model group showed lower attention, as indicated by a lower number of correct responses compared to the control mice (Figure 6A); however, both ADHD/HSP-25 and ADHD/HSP-50 mg/kg groups showed significant increases in the correct responses registered in the Y-maze test. Marble-burying behavior is shown in Figure 6B, and ADHD mice showed greater attentive activities and buried marbles compared to control mice. Both ADHD/HSP-25 and ADHD/HSP-50 mg/kg groups showed significant declines in the count of buried marbles compared to the ADHD control group (Figure 6B).

In the current study, the level of glutamate was increased whereas dopamine was decreased in the ADHD group (Table 1). The glutamate level in mice brains was increased in the ADHD model group, ADHD/HSP-25, and ADHD/HSP-50 groups versus the control group. Hence, treatment with hesperetin did not influence brain glutamate levels (Table 1). Regarding dopamine levels, the ADHD/HSP-25 and ADHD/HSP-50 groups showed significant increases in dopamine levels versus the ADHD model group.

The malondialdehyde level in the ADHD model group was greater than (3.2-fold) the control group. The ADHD/HSP-25 and ADHD/HSP-50 groups displayed lower levels of malondialdehyde versus the ADHD model group (Table 1). The GSH, SIRT1, Nrf2, and HO-1 levels declined in the ADHD model group versus the control group; however, the ADHD/HSP-25 and ADHD/HSP-50 groups showed enhanced SIRT1, Nrf2, and HO-1 levels versus the ADHD model group. Meanwhile, the ADHD/HSP-50 group was able to enhance GSH levels (Table 1). In contrast, NFκB and IL-1β were elevated in the brains of the ADHD group to significant extents. The ADHD/HSP-25 and ADHD/HSP-50 groups displayed low concentrations for these two markers when we set a comparison with the ADHD model group.

**Figure 7:**
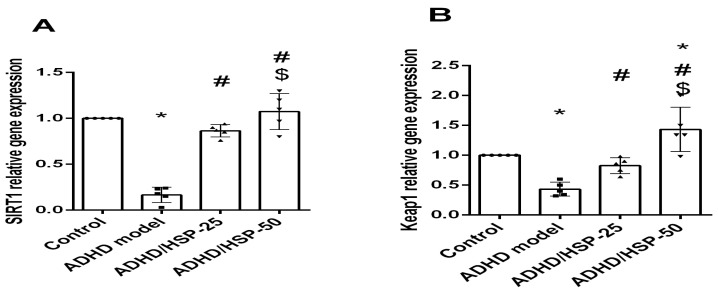

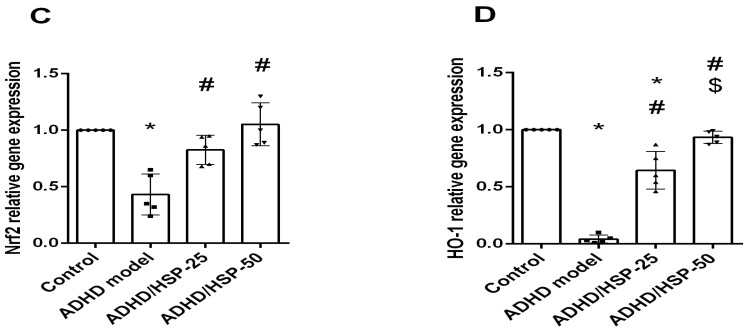
Gene expression analysis for the selected proteins. (**A**) SIRT-1, (**B**) Keap1, (**C**) Nrf2, and (**D**) HO-1 proteins. Data are mean ± SD. *, #, $: Versus control, ADHD model, and ADHD/HSP-25 at *p* < 0.05.

Furthermore, gene expression for SIRT1, keap1, Nrf2, and HO-1 showed a significantly marked reduction in their expression in the ADHD mice group if compared with the control group, and there is a significantly marked restoration of expression levels in the HSP-25 treated mice group with a level of expression approaching that of the control group. Only Keap1 and HO-1 gene expressions showed a significant increase in their expression in HSP-50 treated groups compared to HSP-25, while SIRT1 and Nrf2 showed no significant difference between the HSP-25 and HSP-50 treated groups (Figure 7).

**Figure 8:**
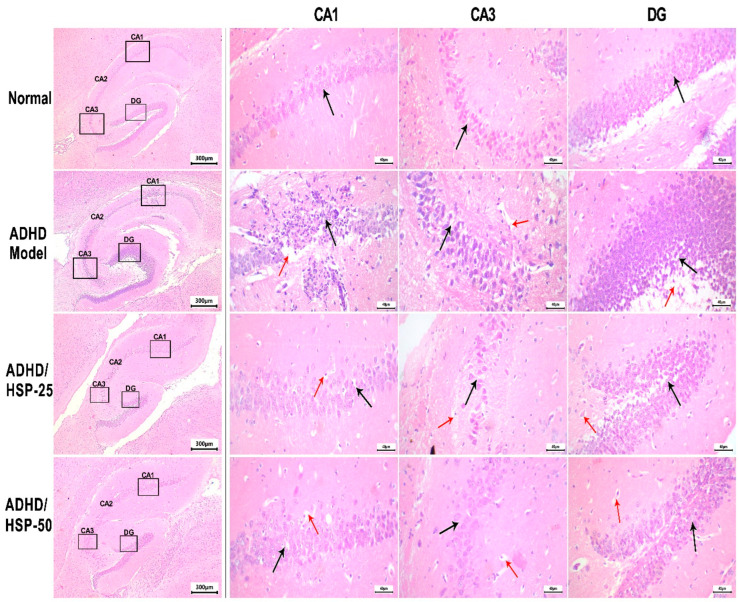
Histopathological image of hippocampal sections stained by hematoxylin and eosin. A photomicrograph of H&E-stained sections in the hippocampus region of the studied groups. Control mice showed small, closely packed neurons and cell bodies, indicated by a black arrow, with intact fibrillary cytoplasmic processes, with thin-walled vessels. ADHD model mice: CA1 region showed marked degenerative changes of neurons with reduced cells, and many neurons showed deeply eosinophilic cytoplasm and dark pyknotic fragmented nuclei (black arrow), with clumping of neuronal processes. There is marked cytoplasmic vacuolation with marge communicating vacuoles (red arrow). CA3 region showed mild pericellular halos (black arrow) with mild degenerative changes, perivascular vacuolation, and fibrillary process fragmentation (red arrow). DG region showed spacing of neuronal cell bodies (black arrow) with marked vacuolation (red arrow). ADHD/HSP-25 mice: The CA1 region showed minimal residual changes in the form of focal pericellular vacuolation (black arrow) and scattered glial cell vacuolation (red arrow). CA3 region showed pericellular vacuolation (black arrow) and focal fibrillary process degeneration (red arrow). DG region showed focal mild neuronal cell body derangement (black arrow) with minimal vacuolation (red arrow). ADHD/HSP-50 mice: CA1 region showed minimal residual changes in the form of minimal focal pericellular vacuolation (black arrow) and scattered glial cell vacuolation (red arrow). CA3 region showed no evidence of vacuolation (black arrow) and focal peri glial vacuolation (red arrow). DG region showed intact neuronal cell bodies with no vacuolation (black arrow), with minimal glial cell vacuoles (red arrow). A low magnification image (40×) on the left identifying different parts of the hippocampus where changes are discussed and magnified (400×) for each group (CA: Cornu Ammonis, CA1 in the superior region, CA3 in the inferior region, DG: Dentate gyrus).

In Figure 8, we can observe a photomicrograph of H&E-stained sections in the hippocampal CA1 region, which was greatly affected by feeding an MSGL diet. Control mice showed small, closely packed neurons and cell bodies indicated by the black arrow, whereas the ADHD model mice showed a CA1 with degenerated neurons with reduced cells, and many neurons showed a deeply eosinophilic cytoplasm and dark pyknotic fragmented nuclei. In addition, the DG region showed the spacing of neuronal cell bodies with marked vacuolation. On the other hand, ADHD/HSP-25 mice showed a CA1 region with minimal residual changes in the form of focal pericellular vacuolation. The DG region showed focal mild neuronal cell body derangement with minimal vacuolation. ADHD/HSP-50 mice showed a CA1 region with minimal residual changes in the form of minimal focal pericellular vacuolation. The CA3 region showed no evidence of vacuolation and focal peri glial vacuolation. The DG region showed intact neuronal cell bodies with no vacuolation (Figure 8).

**Figure 9:**
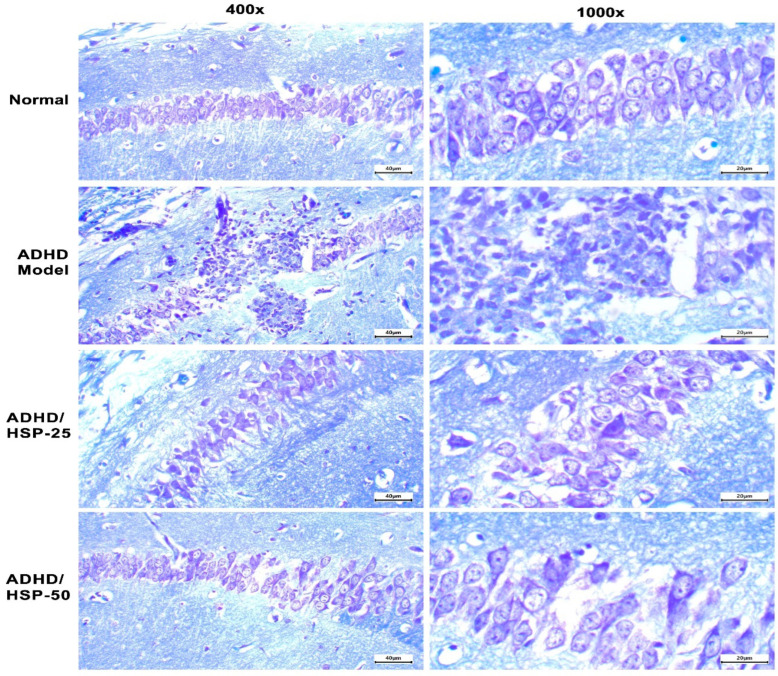
Histopathological image of hippocampal sections stained by toluidine blue stain. Row 1: Control mice fed on the normal diet show normal arrangement with prominent nuclei. Row 2: The ADHD model group shows degenerated neurons and loss of the normal arrangement of the CA1 hippocampal region with the outgrowth of neurons outside the default, arranged packed line of neurons. Rows 3 and 4: Mice in ADHD/HSP-25 and ADHD-50 groups show retainment of the normal packing of hippocampal CA1 neurons and restoration of the nuclear and cytoplasmic structures. Toluidine blue staining (×100 on the left side and ×400 on the right side).

Toluidine blue hippocampal staining indicated that control mice fed on the normal diet showed normal arrangement and appearance with prominent nuclei (Figure 9, Row 1). However, the ADHD model group showed degenerated neurons and a loss of the normal arrangement of the CA1 hippocampal region with the outgrowth of neurons outside the default, arranged packed line of neurons (Figure 9, Row 2). Mice in ADHD/HSP-25 and ADHD-50 groups showed retainment of the normal packing of hippocampal neurons and restoration of the nuclear and cytoplasmic structures (Figure 9, Row 3 and 4).

**Figure 10:**
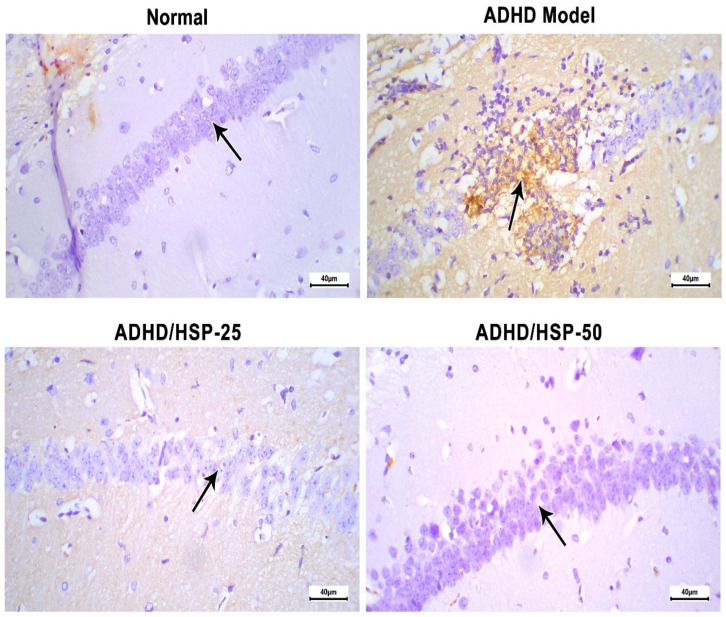
Histopathological images of hippocampal sections immune-stained for IL-1β. Control group: Mice fed on the normal diet show fairly negative staining and normal arrangement with prominent nuclei. ADHD model group: An image shows degenerated neurons with strong immunostaining in the CA1 hippocampal region. ADHD/HSP-25 and ADHD-50 groups: Arrow indicates the immunostaining in the neurons of the CA1 region (superior region of the Cornu Ammonis). Images show a gradual decrease in immunostaining in the CA1 region. Immunostaining (×400).

Further, immunohistochemical staining for IL-1β is shown in Figure 10. The immunostaining in the hippocampal CA1 region of the control group is nearly negative. In contrast, the ADHD model group showed robust staining (Figure 10). The ADHD/HSP-25 and ADHD-50 groups showed weak or very weak staining (Figure 10).

## 3. Discussion

ADHD is a neuropsychiatric disorder affecting children and has few therapeutic options. Various studies indicated that therapeutic interventions may reduce the symptoms of ADHD. In this study, we tested the ability of hesperetin to alleviate experimental ADHD in mice, and the assessment was based on behavioral psychomotor, biochemical, and histopathological investigations.

Pharmacotherapy is very crucial for ADHD, and among individuals diagnosed with ADHD, initiation of medication was found linked to significantly reduced mortality, in particular for death resulting from unnatural causes [25]. The prolonged duration of stimulant use [26] and the increase in the usage of stimulant drugs induced a global concern and resulted in public and political debates [27]. For example, stimulants may affect the appetite and growth of children with ADHD [28] and lead to a reduction in expected height gain [29]. More serious issues with both stimulants and non-stimulants are the possible cardiovascular adverse effects. These drugs may increase the heart rate, blood pressure, and cardiac rhythm, though mostly on a clinically insignificant level [30,31]. All these factors make it necessary to discover new complementary treatments for ADHD with a low incidence of adverse effects.

In the bioinformatic study, we have identified that hesperetin exhibits significant antioxidant and anti-inflammatory effects, specifically within the ROS signaling pathway. Hesperetin appears to modulate the ROS signaling pathway by scavenging free radicals and lowering oxidative stress. This activity helps in mitigating the damage induced by ROS, which are known to contribute to various inflammatory processes and oxidative stress-related diseases. Our findings suggest that hesperetin’s influence on the ROS pathway could make it a promising therapeutic agent for conditions characterized by excessive oxidative stress and inflammation. We have highlighted the role of hesperetin in modulating the Nrf2/Keap1/HO-1 pathway within the ROS signaling cascade. Figure 1 illustrates this interaction and its significance in antioxidant defense mechanisms. Our study suggests that when hesperetin activates the Nrf2/Keap1/HO-1 pathway, this is a key mechanism through which it exerts its protective effects against ROS-induced damage, highlighting its potential as a therapeutic agent in managing oxidative stress-related conditions.

Our STRING db search has identified a strong PPI among the studied proteins involved in the Nrf2/Keap1/HO-1 pathway. The interactions included the co-expression of genes, experimentally determined interactions, and interactions from curated databases and text-mining between Nrf2, Keap, and HO-1. Our enrichment analysis has revealed 96 pathways related to hesperetin target genes and ADHD genes, among which 14 pathways were intercalated, which indicates a strong relation between the two sets of genes. This suggests potential therapeutic implications of hesperetin in the context of ADHD. The pathways involved are likely critical in the modulation of both oxidative stress and neuroinflammatory processes, which are relevant to ADHD pathophysiology. Interestingly, our further analysis discovered the top GO biological processes in relation to hesperetin target genes and the top diseases that are related to hesperetin therapeutic action, which include Alzheimer’s disease and Parkinson’s disease, which have similar cognitive components as ADHD.

In this study, feeding the rats with MSGL induced changes in brain glutamate and dopamine levels, leading to noticeable poor attention and highly increased spontaneous movement. In this study, feeding the rats with MSGL induced memory, locomotor, and behavioral abnormalities in the ADHD group. There was evidently increased spontaneous locomotor activity together with attenuated attention and spatial memory. The hyperkinetic disorder in the ADHD mice was confirmed by the long duration of mobility within the arena of the OFT associated with a larger number of visits to the central zone. There was also an increased number of rears (elevated forelimbs only) and increased number of crossed squares in the ADHD animals compared to the normal values. Additionally, the activity index was also increased. The activity index is an indicator of the distance of locomoting activity and calculated as number of crossed squares/number of stops. Together, these results denote locomotor hyperkinetic disorder, which is characteristic for ADHD.

The ADHD group results also found that there is an increase of the buried marbles in the marble burying test. This indicates compulsive stereotyped behavior; it also an indicator of anxiety behavior and attention deficit [32]. This repetitive compulsive (attention deficit) behavior, together with the decreased number of correct responses in the Y-maze test (which is a specific test for spatial learning and memory), pointed to the observation that the hyperkinetic locomotion observed in the ADHD mice was non-executive and associated with cognitive dysfunction. In agreement, it was reported that ADHD is associated with abnormalities in brain structures such as the unusual development of brain neuronal networks. This includes the prefrontal striatal circuits that mediate cognitive and executive functions [33,34,35].

From our point of view and according to our results, oxidative and inflammatory stresses have contributed to the development of ADHD-related locomotor and behavioral disorders. We observed increased level of MDA (oxidative stress marker) and inflammatory markers in the ADHD group of mice. Oxidative stress was established as a significant contributing factor in the etiology of ADHD [36,37], and the nuclear receptor Nr2f1 of the inflammatory pathway plays important functions in specifying diverse neuron subtypes throughout the patterning of the neocortical motor and somatosensory cortex, in addition to the regulation of the longitudinal hippocampal growth during development [32].

On the other hand, treatment with flavonoid hesperetin ameliorated ADHD-induced behavioral alteration. Hesperetin partially improved spontaneous locomotor activity as well as spatial learning and memory behaviors. The drug administration led to a partial correction of the locomotor hyperkinesia as demonstrated by the decline of the time spent and the number of visits to the central zone of the OFT arena. It also dose-dependently decreased the number of rears, number of crossed squares, and the activity index.

Hesperetin did not only improve the locomotor hyperkinesia but also limited the repetitive compulsive behavior of ADHD and ameliorated attention and cognitive functions. It diminished the number of buried marbles and increased the number of correct responses in the Y-maze test in a dose-dependent fashion. Indeed, restoration of the endogenous antioxidant and decreased neuroinflammatory markers by hesperetin conferred an explanation for this locomotor and behavioral amelioration, though it was incomplete.

In the current study, the histopathological picture of the ADHD group after exposure to MSGL showed disruption of the neuronal arrangement in the hippocampus with smudged nuclei and prominent cytoplasmic processes and vacuolation in the affected neurons. In the cerebral cortex of the ADHD rats, MSGL produced histopathological changes in the cerebral cortices in the form of slightly dense cytoplasm and focal cytoplasmic vacuoles. The neurons of the hippocampus and cerebral cortex showed dense deeply stained cytoplasm with condensed granules by silver stain. These results were in agreement with Owoeye and Salami (2017) who found that MSGL treatment produced degenerated pyknotic neurons in the cerebral cortex and pyknotic neurons and disruption of the normal layers of the neurons in the Cornu Ammonis3 (CA3) of the hippocampus [38]. Another study found that MSGL administration produced degenerative changes in the pyramidal and granule cells of CA1, CA3, and dentate gyrus of the hippocampus [39].

The altered neuronal arrangement and the densely packed cytoplasm could be attributed to the excitotoxic effect of MSGL which leads to apoptosis via increased calcium influx and stimulation of a cascade of intracellular enzymatic reactions. Earlier studies were in agreement with this explanation [39,40].

In the current study, hesperetin ameliorated the histopathological alteration in the hippocampus and the cerebral cortex. Earlier studies proved the neuroprotective effect of hesperetin in multiple neurological disorders [41,42]. Further findings support hesperetin’s protective benefits against Aβ-induced neuroinflammation in in vitro studies, which demonstrated the suppression of TLR4 and p-NF-kB by hesperetin [43]. In the current study, immunohistochemically stained sections from the mice brains exposed to MSGL showed a decreased expression of HO-1 in the nuclei of the hippocampal and cortical neurons. In the present study, hesperetin showed neuroprotection against brain injury induced by the feeding of MSGL. This anti-apoptotic effect of hesperetin agrees with previous results, which highlighted that hesperetin inhibits striatal 6-OHDA lesion-induced apoptosis and decreased neuronal degeneration in Parkinson’s disease [44].

Moreover, SIRT1 speeds up ROS detoxification through the upregulation of cellular antioxidant molecules and enzymes. The current study documented increased MDA levels, which is an indicator of increased oxidation and low SIRT1 expression in the ADHD model group. It was verified that many stressful disorders are probable to lessen SIRT1 levels. It is well-documented that oxidative stress reduces the expression of SIRT1 [45]. Similarly, Kao et al. reported that resveratrol (a SIRT1 activator) increases SIRT1 expression and mitigates ROS production by H_2_O_2_, leading to reduced endothelial cell senescence [46]. The exact mechanism involves a greater levels of ROS exhibiting a significant decline in the glutathione redox capacity inside the cell [47,48]. Additionally, the increase in ROS production activates biochemical cascades that can enhance cellular apoptosis [49].

The SIRT1 cascade is the most extensively studied cascade in the brain, as it is expressed at a higher level [50]. Previous research has shown that mice overexpressing SIRT1 in the brain experience diminished hippocampal damage induced by cerebral ischemia compared to mice lacking SIRT1 expression. This supports the notion that SIRT1 performs an essential role in protecting the brain from damage events, as it is vital to reducing oxidative stress [51,52]. One of the downstream proteins for SIRT1 is Nrf2, which provides additional defense against oxidative stress damage. One study conducted by Liao et al. demonstrated that Nrf2 knockout mice can develop depressive-like behavior [53].

There is a cross-link between the Keap1 and Nrf2 proteins [54]. In the absence of stress, Keap1 enhances the ubiquitination of Nrf2, promoting its degradation and suppressing its transcriptional activity. Nonetheless, during stress conditions, the cysteine residues of Keap1 are modified to prevent Nrf2 degradation, allowing it to accumulate and induce antioxidant genes [55]. Furthermore, the presence of Nrf2 attenuates the expression of chemokine-related genes that can suppress the inflammatory process [56]. There is also a close link between the Nrf2/HO-1 pathway in neurological conditions like major depression disorder [57]. Moreover, Nrf2/HO-1 dysfunction reduces the level of antioxidant enzymes after an event of oxidative stress, and this was shown to amend the risk of associated neurologic injury [58]. Further, losing Nrf2/HO-1 results in a decline in the expression of HO-1 protein and an increase in oxidative damage, and this can be a risk for increasing neurological impairment, as shown in ischemic stroke [59].

As oxidative stress and inflammation are known to have a role in the incidence of neurodegenerative diseases, the SIRT1/Nrf2/HO-1 pathway possesses an attractive target for the treatment of ADHD diseases. Indeed, a previous study demonstrated the anti-inflammatory effect of hesperetin in decreasing the over-expression of inflammatory cytokines (IL-1β, TNF-α, and IL-6) and mitigating the activation of NFκB in lipopolysaccharide-stimulated BV-2 microglial cells and in the lipopolysaccharide-challenged mouse brain [60]. In agreement with our results, hesperetin overcomes ROS production and stimulates Nrf2 and HO-1 in mice brains after exposure to lipopolysaccharides. Another in vitro study reported a cytoprotective effect for hesperetin against lipopolysaccharide-mediated oxidative stress in the HT-22 cell line, which are mouse hippocampal neurons [61].

In the current study, hesperetin mitigated hippocampal neuron damage induced by MSGL in the ADHD model group. Similarly, a previous study highlighted that hesperetin provides protection to hippocampal neurons in mice exposed to Aflatoxin B1 and was able to restore GSH interrupted by Aflatoxin B1 [62]. Song et al. documented that hesperetin improves neurobehavioral function due to traumatic brain injury by limiting microglial activation and the subsequent inflammatory burden via the AMPK-SIRT1-FoxO1-NF-κB axis [63].

Consistently, flavonoids (such as hesperetin in our model) are widely used in the treatment of different neurological disorders, and they mainly act through their antioxidant potential, as well as expanding the available dopamine. However, therapeutic interventions with hesperetin or a hesperetin-rich diet can be suggested as a complementary treatment in ADHD patients but cannot be recommended as an ADHD treatment per se as it is a heterogenous and complex disease [64].

In conclusion, hesperetin was proven useful as a therapeutic tool for alleviating psychomotor symptoms, upregulating SIRT1/Nrf2/Keap1/OH-1 proteins, and downregulating NFκB/IL-1β signaling; all these outcomes were accompanied by a significant restoration in hippocampal neurons. All these benefits may suggest hesperetin as a dietary component for ADHD children until sufficient studies are conducted for a full exploration of the mechanism of action and clinical utility.

The current study considered hesperetin as a safe remedy and has no per se effect on mice based on a previous report [65]. Hence, the experimental design did not include a hesperetin-only group. This is one of the limitations of this study. In our experimental design, hesperetin was administered from the 5th week of MSGL administration; in this way, it was neither a preventive effect nor a typical reversal of ADHD symptoms or treatment. Hence, it will be recommended to design future experiments based on a preventive schedule starting from day 1 of MSGL.

## 4. Materials and Methods

### 4.1. The Bioinformatic Study

We investigated the KEGG database to look for the hesperetin mechanism of action pathway as an antioxidant and anti-inflammatory drug on 30 April 2024. The studied proteins (Nrf2/kelch-like ECH-associated protein 1 (Keap1)/hemeoxygenase-1) were entered in the search tool one by one and the common pathways were retrieved. Then, we utilized version 12 of the STRING database (https://string-db.org/) to discover PPI among different studied proteins (Nrf2/keap1/HO-1) on the 1st of May 2024. The studied proteins were entered under “Multiple Proteins” search, with Homo sapiens as the organism. In addition, we used PubChem and Drugbank db to retrieve hesperetin targets on 2 May 2024. On the same day, the DisGeNet database was used to find genes related to ADHD. Over-representation analysis (ORA) was performed to identify diseases significantly associated with our gene list. We utilized a *p*-value threshold of 0.05 and applied FDR correction with a threshold of 0.05 to account for multiple testing. Only associations with a DisGeNET GDA score above 0.4 were considered to ensure high confidence in the results. Functional enrichment analysis and visualization for the interactions between hesperetin and ADHD were conducted using ShinyGo 0.80 and FunRich bioinformatic tools.

### 4.2. The Mouse Study

#### 4.2.1. Chemicals

Hesperetin was procured from Sigma-Aldrich Company (St. Louis, MO, USA) and suspended in a 1% carboxymethylcellulose (CMC) solution, and a stock preparation was aliquoted in 5 mL samples in Eppendorf tubes at −4 °C as instructed by the suppliers. AL-Gomhoria Company supplied monosodium glutamate (MSGL) (Cairo, Egypt). Daily preparation of MSGL included 0.4 g/kg in a standard mouse chow diet for 8 weeks [66].

#### 4.2.2. Animals

Male Swiss albino mice (n = 24, postnatal day 30, weighing 15–18 g) were obtained from the Abu-Rawash Company (Cairo, Egypt) and housed in hygienically controlled laboratory conditions and a normal dark/light cycle. Mice were housed in groups of six in polyethylene cages (38 × 25 × 22 cm). Mice had free access to the diet (normal chow diet or MSGL diet) and tap water throughout the experiment. The study protocol obtained approval number 5690# from the Research Ethics Committee of the Faculty of Medicine at Suez Canal University and number 202405RA1 from the Ethics Committee of the Suez Canal University Faculty of Pharmacy. The work met the terms of the Guide for the Care and Use of Laboratory Animals announced by the NIH (NIH Publications No. 8023, revised 1978).

#### 4.2.3. The Design of the Experiment

**Figure 11:**
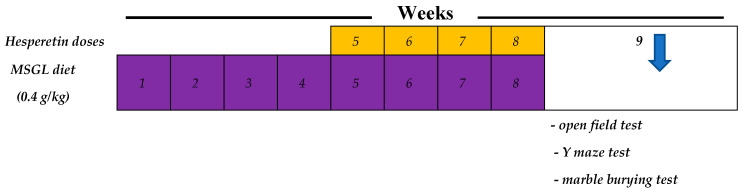
The time course of the experiment and behavior tests.

Mice were allocated into four groups (Figure 11) of six mice each as follows: Group I mice served as normal control and received a normal chow diet and administered distilled water only. Group II served as ADHD control animals and received MSGL diet (0.4 g/kg) [66] and administered oral doses of 1% CMC solution (parallel to hesperetin). Group III and IV received a 0.4 g/kg MSGL diet and administered HSP-25 and HSP-50 mg/kg by oral gavage in a volume equal to 0.2 mL/mouse [67]. Mice were exposed to behavioral tests the next day after finishing the experiment.

#### 4.2.4. Behavioral Assessments

##### The Open Field Test (OFT)

The apparatus was composed of an opaque acrylic container—each side measured 40 cm in height and 80 cm in length, and the floor was divided into 16 equal-sized squares, as previously mentioned by [68]. Each mouse was placed in the apparatus center. The behavioral traits for locomotor activity were examined by measuring the central zone duration (time spent in the outlined zone), count of entries to the central zone, rearing, mobility time, crossed squares, and activity index. The activity index was calculated for each animal by dividing the square numbers by the stop numbers in 3 min [69]. For every trial, every animal was situated in the middle of the field. Before the behavioral testing, the open field was cleaned with a 5% water-ethanol solution to avoid any possible discrimination related to scents left by prior mice [70].

##### Test of Y-Maze Discrimination Learning

The Y-maze discrimination learning test was performed following Xu’s method [71]. For six consecutive days, mice performed a Y-maze discriminating learning test in the last week of the experiment. The outcomes were recorded as correct responses for 20 responses and expressed as mean ± SD [72].

##### Marble Burying Test (MBT)

MBT was utilized to evaluate the mice’s compulsive behavior. The test cages were set up with 15 glass marbles (15 mm diameter) uniformly arranged in three rows on 5 cm of clean bedding in each cage. In this experiment, a mouse was set up in a typical Plexiglas test cage under lighting sites that were identical to those in the animal facility. After 20 min of assessment, the number of marbles buried (with at least 2/3 covered by bedding) was recorded [73].

#### 4.2.5. Histopathological Examination and Photomicrography

Mice were euthanized by an intraperitoneal dose of ketamine (80 mg/kg) and sacrificed via dislocating the cervical vertebrae, and then the brains were immediately removed on an ice-cold plate and cut into two halves. For biochemical analysis, the first portion was kept at −80 °C, while the second portion was fixed in 10% formalin and utilized for histopathology examination. Formalin-fixed brain hemispheres were inserted in paraffin wax, and then 4 μm sections were prepared. We outlined the location of the hippocampal region according to the Mouse Brain Stereotaxic Atlas. Three brain slices were prepared at the area of the largest hippocampal section as detected with the naked eye. Hematoxylin and eosin routine staining (H&E) was performed. An experienced pathologist investigated the slides for histopathological changes in a blinded way. Some criteria were set for investigation, including the presence of eosinophilic foci of degenerated neurons, vacuolation, and the degree of gliosis [74]. Images were obtained at 40× and 400× magnification.

In addition, another section from the mice’s brains was stained with toluidine blue stain to assess the integrity of the neurons. It is a basic thiazine dye that has an affinity to the basophilic tissue elements and hence has the ability to stain tissues rich in DNA and RNA. It is widely used as a special stain owing to its metachromatic properties. Toluidine blue staining was performed as previously described [75].

#### 4.2.6. Immunohistochemical Assessment of IL-1β Protein

Staining for IL-1β in specimens was assessed immunohistochemically. We prepared 5 μm thick sections and exposed them to heating for 12 h in an oven to achieve sufficient adhesion. Then, sections were deparaffinized, rehydrated, and antigen retrieved by exposure to irradiation in a microwave while covered with 0.01 M citrate buffer (pH = 6) and heated at the microwave high power for 15 min. After that, sections were put in a humid chamber and incubated with the primary rabbit polyclonal antibody to mouse IL-1β (ThermoScientific, Fremont, CA, USA). After washing, staining was completed using Mouse/Rabbit PolyDetector, BioSB (Goleta, CA, USA) kit. We applied the horseradish peroxidase label (HRP) and 3,3′-diaminobenzidine, then used Meyer’s hematoxylin for counterstaining. Then, three fields from each slide were examined and imaged at 400×. Immunohistochemistry was assessed utilizing the ImageJ 1.45 software (NIH, Bethesda, MD, USA) to determine the area of immunostaining [76,77].

#### 4.2.7. Measuring of Whole Brain Oxidative Stress Parameters

The frozen brains were homogenized for estimating malondialdehyde (MDA) using the thiobarbituric acid method [78,79]. In addition, reduced glutathione was measured in the brain homogenate using a previously designated method, in which GSH will reduce 5,5′-dithiobis(2-nitrobenzoic acid), producing a yellow product. The chromogen is in direct proportion to GSH and was measured at 405 nm [78]. Kits were bought from BioDiagnostics (Cairo, Egypt).

#### 4.2.8. Enzyme-Linked Immunosorbent Assays (ELISA)

By using mice enzyme-linked immunosorbent test (ELISA) kits, the following biomarkers were evaluated in 10% brain supernatants: Nrf2 (MBS744301), SIRT (MBS775316, biotin double antibody sandwich technology ELISA), OH-1 (MBS700767), and NFκB (Cat# MBS043224) kits obtained from MyBioSource (San Diego, CA, USA), and IL-1β (Cat# E-EL-M0037, Elabscience, Houston, TX, USA) kits according to the manufacturer’s instructions. In addition, dopamine (Cat# MBS732020) and glutamate (Cat# MBS756400) ELISA kits were used. The measurements were performed employing an automated ELISA reader. The color depth or light was positively correlated with the concentration of the measured proteins.

#### 4.2.9. Gene Expression Measurement of Nrf2, Keap1, SIRT1 and Hemoxygenase-1 by Quantitative Real-Time PCR (qPCR) Analysis

Firstly, miRNeasy Mini Kit (CAT. NO. 217004 QIAGEN GmbH, Hilden, Germany) was used to extract the total RNA from homogenizing brain tissues (25 mg of tissues/mouse). Then, the cDNA was formed from the extracted RNA using High-Capacity Reverse Transcriptase kits (cat. no. 4368814) from Thermos Fisher Scientific company, Winsford, UK. Finally, the relative quantitative measurement of SIRT1, Nrf2, Keap1, and OH-1 genes was performed using ready-to-use PowerTrack SYBR Green (cat.no. A46109) from Thermos Fisher Scientific company, Winsford, UK, and the specific primers used, as shown in Table 2, are from OriGene Global company, Rockville, MD, USA.

The PCR reaction was computed on a StepOne real-time PCR instrument (cat. no. 4376357, Thermo Fisher Scientific, Altrincham, UK) by adjusting the instrument first at 95 °C for 5 min. This was followed by 35 cycles of 95 °C for 15 s, an annealing temperature of 60 °C for 1 min, and 72 °C for 30 s. After obtaining results from the instrument, the gene expression was estimated by extracting the cycle threshold (CT) of target genes from that of the housekeeping gene (β-actin gene) in different experimental groups and the control group, then the 2^−ΔΔCt^ equation reported by Livak & Schmittgen [79] was used to calculate the fold change in experimental groups.

#### 4.2.10. Statistical Analysis and Data Presentation

The data is described as mean ± SD and checked for normality by using the Kolmogorov–Smirnov (KS) test. Data that displayed normal distribution were evaluated (at *p* < 0.05) by applying one-way ANOVA tests and Tukey’s post-hoc tests. The GraphPad Prism software version 9 (Graph Pad Software, Inc., San Diego, CA, USA) was utilized.

## Figures and Tables

**Table 1:** Effect of hesperetin on the level of dopamine, glutamate, and oxidation/inflammation markers in brain homogenates.

Markers	Control	ADHD Model	ADHD/HSP-25	ADHD/HSP-50
Glutamate (ng/g)	11.3 ± 1.28	42.04 ± 5.07 *	38.6 ± 4.56 *	42.6 ± 5.94 *
Dopamine (ng/g)	25.62 ± 3.94	7.38 ± 1.63 *	12.12 ± 1.46 *#	18.32 ± 1.99 *#
Malondialdehyde	6.26 ± 0.78	20.4 ± 2.78 *	11.96 ± 2.04 *#	7.98 ± 0.69 #
Reduced glutathione	90.8 ± 14.65	35.4 ± 9.45 *	44.4 ± 9.37 *	67.8 ± 10.80 *#$
SIRT1 (ng/g)	12.25 ± 2.32	2.6 ± 0.82 *	6.06 ± 1.06 #	8.56 ± 1.77 *#
Nrf2 (ng/g)	66.4 ± 6.11	15.6 ± 5.77 *	28.6 ± 15.95 *#	46.2 ± 7.05 *#$
HO-1 (pg/g)	206.2 ± 15.06	48.6 ± 20.79 *	116.2 ± 16.12 *#	156.2 ± 20.54 *#$
NFκB (pg/g)	64.4 ± 16.73	482.8 ± 66.15 *	404 ± 44.07 *#	238.2 ± 42.90 *#$
IL-1β (pg/g)	17.4 ± 3.21	74.2 ± 8.26 *	51.4 ± 4.39 *#	31.8 ± 5.72 *#$

Data are mean ± SD. *, #, $: Versus control, ADHD model, and ADHD/HSP-25 at *p* < 0.05.

**Table 2:** Target and housekeeping gene primer sequences.

The Target Gene	Primers	Accession Numbers
*SIRT1*	F: 5′-GGAGCAGATTAGTAAGCGGCTTG-3′ R: 5′-GTTACTGCCACAGGAACTAGAGG-3′	NM_019812
*Keap1*	F: 5′-ATCCAGAGAGGAATGAGTGGCG-3′ R: 5′-TCAACTGGTCCTGCCCATCGTA-3′	NM_001110305
*β-actin*	F: 5′-CATTGCTGACAGGATGCAGAAGG-3′ R: 5′-TGCTGGAAGGTGGACAGTGAGG-3′	NM_007393
*HO-1*	F: 5′-CCAGGCAGAGAATGCTGAGTTC-3′ R: 5′-AAGACTGGGCTCTCCTTGTTGC-3′	NM_002133
*Nrf2*	F: 5′-CAGCATAGAGCAGGACATGGAG-3′ R: 5′-GAACAGCGGTAGTATCAGCCAG-3′	NM_010902

## Data Availability

Data is contained within the article.
